# Using penalized regression to predict phenotype from SNP data

**DOI:** 10.1186/s12919-018-0149-2

**Published:** 2018-09-17

**Authors:** Svetlana Cherlin, Richard A. J. Howey, Heather J. Cordell

**Affiliations:** Institute of Genetic Medicine, Newcastle University, International Centre for Life, Central Parkway, Newcastle upon Tyne, NE1 3BZ UK

## Abstract

**Background:**

In a typical genome-enabled prediction problem there are many more predictor variables than response variables. This prohibits the application of multiple linear regression, because the unique ordinary least squares estimators of the regression coefficients are not defined. To overcome this problem, penalized regression methods have been proposed, aiming at shrinking the coefficients toward zero.

**Methods:**

We explore prediction of phenotype from single nucleotide polymorphism (SNP) data in the GAW20 data set using a penalized regression approach (LASSO [least absolute shrinkage and selection operator] regression). We use 10-fold cross-validation to assess predictive performance and 10-fold nested cross-validation to specify a penalty parameter.

**Results:**

By analyzing approximately 600,000 SNPs we find that, when the sample size comprises a few hundred individuals, SNP effects are heavily penalized, resulting in a poor predictive performance. Increasing the sample size to a few thousand individuals results in a much smaller penalization of the true effects, thus greatly improving the prediction.

**Conclusions:**

LASSO regression results in a heavy shrinkage of the regression coefficients, and also requires large sample sizes (several thousand individuals) to achieve good prediction.

## Background

In a typical genome-wide association study (GWAS), several thousands to several millions of single nucleotide polymorphism (SNP) markers are genotyped in a sample size of several hundred to several thousand individuals, thus leading to many more predictor variables than response variables. In this case, multiple linear regression cannot be used because the unique ordinary least squares estimators of the regression coefficients are not defined. Methods that allow for more predictors than observations [1] may cause model overfitting. Overfitted models are likely to demonstrate poor predictive ability when applied to new data. To overcome these problems, penalized regression methods have been proposed [2–6], aiming at shrinking the regression coefficients toward zero. Depending on the form of the penalty function, some methods (eg, ridge regression [3]) only shrink the coefficients without setting them to zero, whereas other methods (eg, the least absolute shrinkage and selection operator [LASSO] regression [2]) allow shrinkage of the coefficients down to exactly zero, thus performing variable selection.

The strength of the penalty is controlled by a regularization parameter that determines the amount of shrinkage imposed. One challenge of penalized approaches is choosing an optimal value of the regularization parameter. This is often done by *k*-fold cross-validation to find the parameter value in the training folds that minimizes the average mean squared error in the test folds. Assessing the predictive performance can also be done using *k*-fold cross-validation. In this case, the two cross-validation experiments are combined into one so-called nested cross-validation. In nested cross-validation, an outer cross-validation loop is used to assess the predictive performance, while, within each outer fold, an inner cross-validation loop is used to find the regularization parameter [7]. The most commonly used number of inner and outer folds is 10 because it provides a reasonable classification accuracy [8].

Here, we focus on LASSO linear regression, which has the property of variable selection. We apply 10-fold cross-validation to assess the out-of-sample predictive performance, using 10-fold nested cross-validation to specify the penalty parameter. We explore the effect of the sample size on the predictive ability of LASSO regression.

## Methods

The GAW20 data are based on the Genetics of Lipid Lowering Drugs and Diet Network (GOLDN) study data set [9] that investigated the epigenetic determinants of triglyceride (TG) response. TGs are major blood lipids [10] that constitute an important biomarker of cardiovascular disease risk [11]. Previous GWAS studies found a number of loci associated with TG levels [12]. We focus on predicting the TG response by analyzing the GWAS (SNP) data and four measures of TG. The first two measures (at visits 1 and 2) were taken before the lipid-lowering drug treatment; the second two measures (at visits 3 and 4) were taken after the treatment.

We performed quality control (QC) on the GAW20 GWAS (SNP) data using standard procedures outlined in Turner et al. [13]. SNP-level QC removes 63,907 SNPs with low minor allele frequency (< 0.01), and 2694 SNPs for failing a test of Hardy-Weinberg equilibrium (*p* ≤ 0.00001). Of 822 individuals for whom we have SNP data, the simulated phenotype is available for 680 individuals and the real phenotype is available for 778 individuals. Working on a log scale, we take the mean of the TG measures for visits 1 and 2 as a baseline measure, and the mean of visits 3 and 4 as a follow-up measure. If the measure for either visit 1 or 2 is not available, we take the only available measure as a baseline measure. Similarly, if the measure for either visit 3 or 4 is not available, we take the only available measure as a follow-up measure. We adjust the follow-up measure for the baseline measure, age, center, smoking status, and first 20 principal components (PCs) of SNP effects using a linear regression. The number of PCs was defined by examining the quantile–quantile (Q-Q) plot of the *p* values from the ordinary linear regression. When 20 PCs were incorporated into the linear regression, the Q-Q plot showed no inflated *p* values, which suggests that the relatedness and population stratification are accounted for. We take the standardized residuals as our final phenotype.

### Lasso

Consider a standard multiple linear regression, ***y*** = *β*_0_ + ***Xβ*** + ***ϵ***, where ***y*** is a vector of response variables; ***X*** is a *n* × *p* matrix of predictor variables; *β*_0_ is an intercept; ***β =*** (*β*_1_, …, *β*_*p*_) is a vector of regression coefficients; and ***ϵ*** is a vector of the error terms, ***ϵ***~N(0, *σ*^2^). For *n* > *p* the estimated values of the coefficients are found by minimizing the residual sum of squares:

$$ {\widehat{\beta}}_o,\widehat{\boldsymbol{\beta}}=\mathrm{argmin}\ \left[{\sum}_{i=1}^n{\left({y}_i-{\beta}_o-{\sum}_{j=1}^p{\beta}_j{X}_{ij}\right)}^2\right] $$

However, in a typical GWAS, *n* < *p*. In this case, penalized regression is often used, where the estimators of ***β*** are found by minimizing the sum of the residual sum of squares and a penalty function:

$$ {\widehat{\beta}}_o,\widehat{\boldsymbol{\beta}}=\mathrm{argmin}\ \left[{\sum}_{i=1}^n{\left({y}_i-{\beta}_o-{\sum}_{j=1}^p{\beta}_j{X}_{ij}\right)}^2+\lambda P\left(\lambda, \boldsymbol{\beta} \right)\right] $$

Here *P*(*λ*, ***β***) is the penalty function with a regularization parameter *λ* which controls the amount of shrinkage. The LASSO penalty [2] utilizes an *ℓ*_1_-norm penalty, that is, $P\left(\lambda, \boldsymbol{\beta} \right)=\left\Vert \boldsymbol{\beta} \right\Vert {}_{\ell_1}$; consequently, the estimators of the coefficients take the form:

$$ {\widehat{\beta}}_o,\widehat{\boldsymbol{\beta}}=\mathrm{argmin}\ \left[{\sum}_{i=1}^n{\left({y}_i-{\beta}_o-{\sum}_{j=1}^p{\beta}_j{X}_{ij}\right)}^2+\lambda {\sum}_{j=1}^p|{\beta}_j|\right] $$

One important property of the LASSO penalty is that it allows the coefficients to be set to exactly zero, thus performing variable selection.

## Results

### Simulated data

**Fig. 1:**
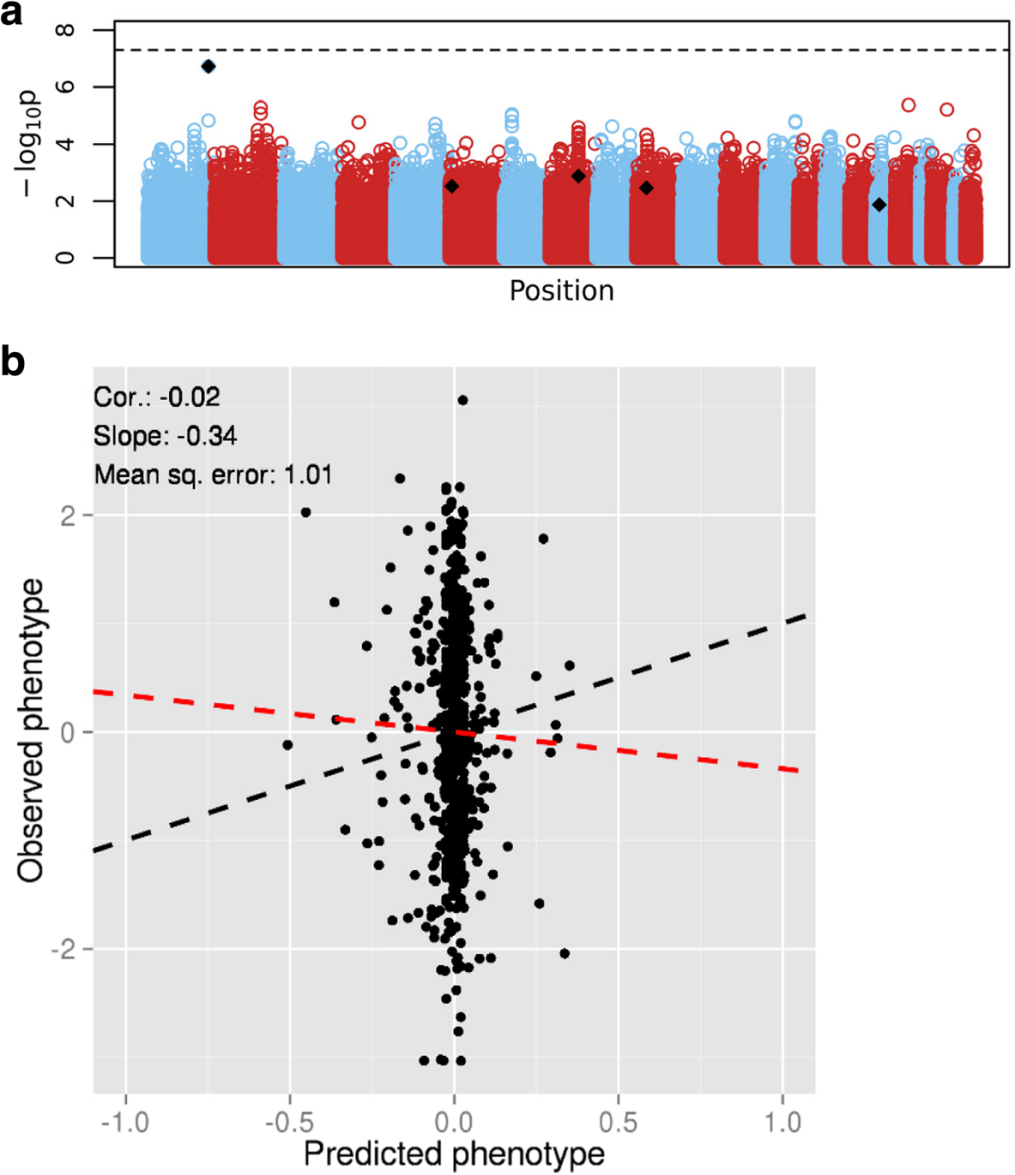
Analysis of the GAW20 simulated data, replicate 84. **a**, Manhattan plot of *p* values from tests of association between SNP and phenotype. The *black dots* represent causal SNPs. The *dashed line* represents genome-wide significance. **b**, Prediction results. The *black dashed line* is the equality line; the *red dashed line* is the best-fit line

We analyzed replicate 84 of the simulated data, consulting the “answers” before performing the analysis. The Manhattan plot from standard linear regression shows no genome-wide significant associations, however, one of the known causal SNPs is nearly genome-wide significant (Fig. 1a). We applied LASSO regression on these data and assessed the predictive performance through 10-fold nested cross-validation, after reducing the number of SNPs to approximately 56,000 using a linkage disequilibrium (LD)–based clumping procedure implemented in the PLINK software [14]. This procedure groups correlated SNPs together and chooses one representative SNP per group, thus reducing dimensionality and eliminating the redundancy of information in the data. We assessed the prediction accuracy through the Pearson correlation coefficient (the square root of heritability), the mean squared error (lower values indicate better fit), and the slope of the best-fit line (a slope of 1 suggests perfect prediction). Figure 1b shows that the predicted phenotypes are heavily shrunk toward zero, resulting in a very low correlation between observed and predicted values and a slope that is far from 1. Poor prediction can be explained by the fact that the effect (regression coefficient) of the most significant true causal SNP is heavily shrunk (a mean estimate of − 0.051 and a SD of 0.062 over the 10 folds, compared to a mean estimate of − 0.443 and a SD of 0.02 over the 10 folds in standard linear regression).

**Fig. 2:**
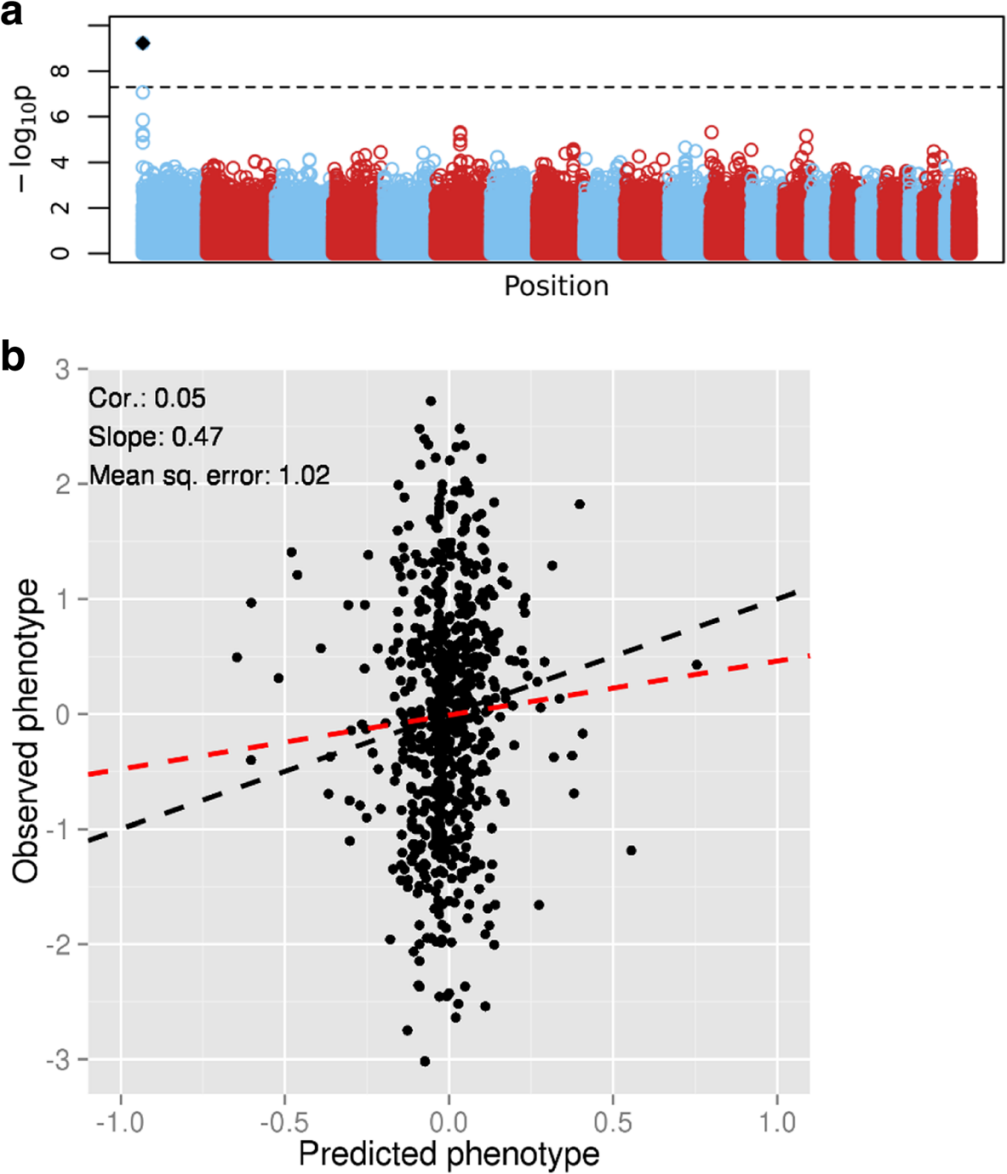
Analysis of the subset of the illustrative simulated data set (700 individuals). **a**, Manhattan plot of *p* values from tests of association between SNP and phenotype. The *black dot* represents the causal SNP. The *dashed line* represents genome-wide significance. **b**, Prediction results. The *black dashed line* is the equality line; the *red dashed line* is the best-fit line

**Fig. 3:**
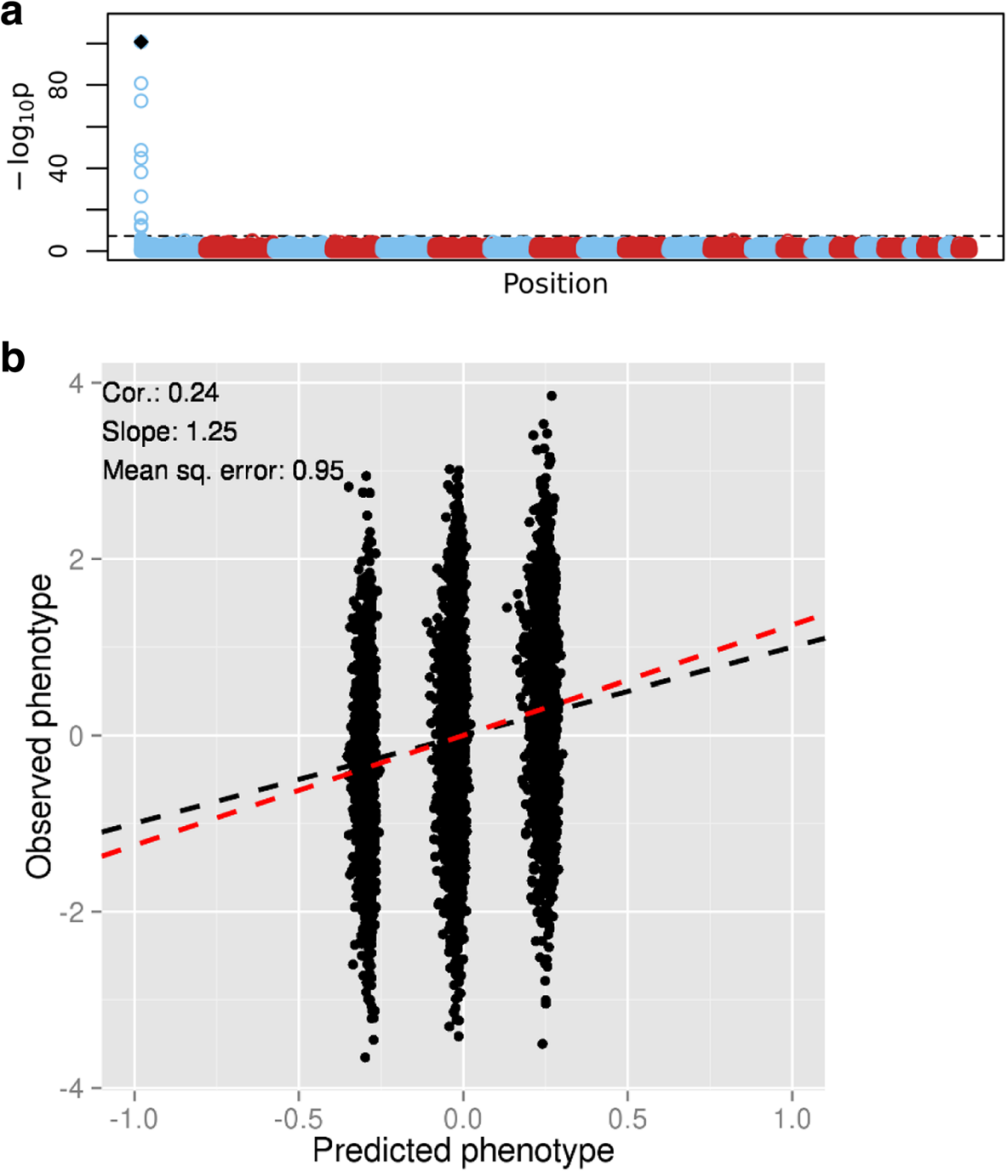
Analysis of the illustrative simulated data set (7753 individuals). **a**, Manhattan plot of *p* values from tests of association between SNP and phenotype. The *black dot* represents the causal SNP. The *dashed line* represents genome-wide significance. **b**, Prediction results. The *black dashed line* is the equality line; the *red dashed line* is the best-fit line

To investigate whether increasing the sample size can improve the prediction, we simulated phenotypes for 7753 individuals for whom we had SNP genotype data available from previous studies. The phenotypes were simulated using only 1 causal SNP with an effect size of − 0.44 (similar to the effect of the nearly genome-wide significant causal SNP from the GAW20 simulated data) and heritability of 0.05, which induces a correlation of approximately 0.23 between the observed and the predicted phenotype. First, we analyzed a subset of these data comprising 700 individuals (similar to the sample size of the GAW20 simulated data). The results resemble those seen in the GAW20 simulated data (Fig. 2). Even though the causal SNP is consistently picked up by LASSO, its effect is poorly estimated (the mean estimate is − 0.096 and the SD is 0.019 over the 10 folds) and has the same order of magnitude as the SNPs (false positive) chosen by LASSO, resulting in poor prediction. We then repeat the analysis using the full sample (7753 individuals). The Manhattan plot shows that the significance of the causal SNP has greatly increased (Fig. 3a). The prediction plots show three distinct clusters representing the estimated effects within the three genotype categories of the causal SNP (Fig. 3b). The effect of the causal SNP is much better estimated (the mean estimate is − 0.247 and the SD is 0.009 over the 10 folds) and the effect size is 10 to 1000 times greater than that of the non-causal SNPs falsely chosen by LASSO. The range of the regularization parameter across folds for the full data set (0.046 to 0.056) is smaller than that for the subset (0.081 to 0.183). This suggests that the regression coefficients for the full data set are much less penalized, greatly improving the prediction.

### Real data

**Fig. 4:**
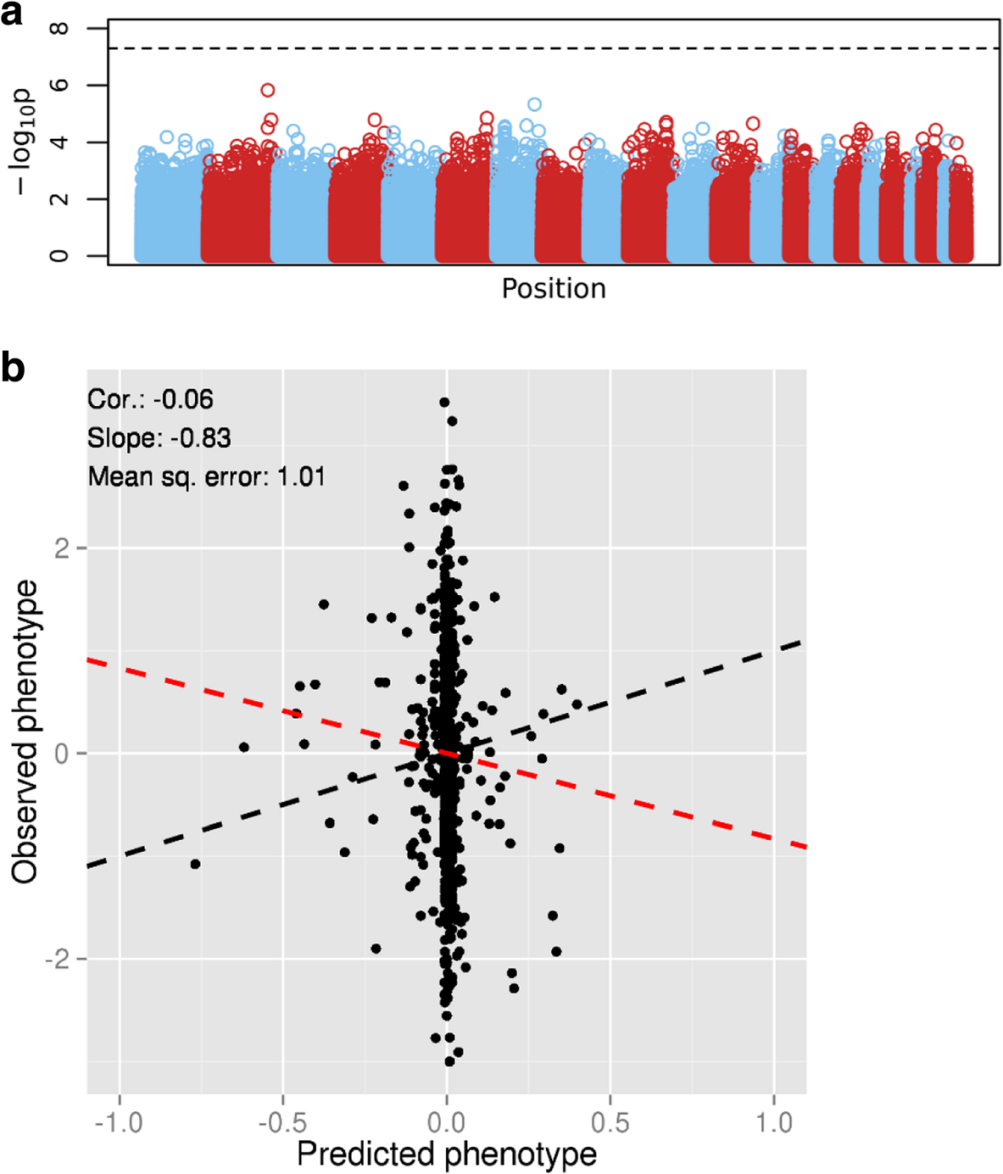
Analysis of the GAW20 real data set. **a**, Manhattan plot of *p* values from tests of association between SNP and phenotype. The *dashed line* represents genome-wide significance. **b**, Prediction results. The *black dashed line* is the equality line; the *red dashed line* is the best-fit line

We also applied LASSO regression to the real GAW20 data and assessed the predictive performance through 10-fold nested cross-validation, after again reducing the number of SNPs to approximately 56,000 using an LD-based clumping procedure. The Manhattan plot shows no genome-wide significant associations (Fig. 4a) and it resembles the Manhattan plot of the GAW20 simulated data. The prediction results are very similar to the results seen in the GAW20 simulated data (Fig. 4b). In a sense, these results are not surprising given the similar sample sizes of the simulated and the real data sets.

## Discussion

The prediction ability of LASSO was assessed on the simulated and the real GAW20 data sets through 10-fold nested cross-validation. Both data sets demonstrated poor predictive performance and exhibited a noticeable shrinkage of the predicted phenotype toward zero. By examining the effect of the most significant causal SNP in the simulated data, we found that it was heavily penalized. To investigate this issue, we analyzed a much larger data set (approximately 7000 individuals). We found that the effect of the causal SNP was much less penalized, thus enabling the best prediction possible with that SNP.

## Conclusions

The prediction ability of LASSO was assessed on the GAW20 data sets and on the much larger data set available from previous studies. Poor predictive performance is achieved for data sets of a few hundred individuals with a weak signal. This can be explained by the fact that the LASSO regression coefficients are substantially shrunk. Other regularized methods that do not result in such a heavy shrinkage of the regression coefficients might be of use. For example, with hyper-LASSO [6] the extent of the shrinkage depends on the size of the coefficients, and adaptive LASSO [5] uses different adaptive weights for penalizing different coefficients. Both of these can potentially lead to a moderate shrinkage. However, with LASSO, increasing the sample size from a few hundred to a few thousand individuals increased the strength of the signal and reduced the amount of shrinkage of the regression coefficients, thus improving the prediction. We conclude that LASSO regression requires large sample sizes (several thousands of individuals) to achieve good prediction.

## References

[1] Efron B, Hastie T, Johnstone I, Tibshirani R (2004). Least angle regression. Ann Stat.

[2] Tibshirani R. Regression shrinkage and selection via the lasso. J R Stat Soc Series B Stat Methodol. 1996;58(1):267–88.

[3] Cessie SL, Houwelingen JCV (1992). Ridge estimator in logistic regression. J R Stat Soc Ser C Appl Stat.

[4] Zou H, Hastie T (2005). Regularization and variable selection via the elastic net. J R Stat Soc Series B Stat Methodol.

[5] Zou H (2006). The adaptive lasso and its oracle properties. J Am Stat Assoc.

[6] Hoggart CJ, Whittaker JC, De Iorio M, Balding DJ (2008). Simultaneous analysis of all SNPs in genome-wide and re-sequencing association studies. PLoS Genet.

[7] Varma S, Simon R (2006). Bias in error estimation when using cross-validation for model selection. BMC Bioinformatics.

[8] Kohavi R. A study of cross-validation and bootstrap for accuracy estimation and model selection. IJCAI-95. 1995:1137–43.

[9] Irvin MR, Zhi D, Joehanes R, Mendelson M, Aslibekyan S, Claas SA, Thibeault KS, Patel N, Day K, Jones LW (2014). Epigenome-wide association study of fasting blood lipids in the genetics of lipid- lowering drugs and diet network study. Circulation.

[10] Kwiterovich PO (2000). The metabolic pathways of high-density lipoprotein, low-density lipoprotein, and triglycerides: a current review. Am J Cardiol.

[11] Miller M, Stone NJ, Ballantyne C, Bittner V, Criqui MH, Ginsberg HN, Goldberg AC, Howard WJ, Jacobson MS, Kris-Etherton PM (2011). Triglycerides and cardiovascular disease: a scientific statement from the American Heart Association. Circulation.

[12] Willer CJ, Schmidt EM, Sengupta S, Peloso GM, Gustafsson S, Kanoni S, Ganna A, Chen J, Buchkovich ML, Mora S (2013). Discovery and refinement of loci associated with lipid levels. Nat Genet.

[13] Turner S, Armstrong LL, Bradford Y, Carlson CS, Crawford DC, Crenshaw AT, de Andrade M, Doheny KF, Haines JL, Hayes G, et al.: Quality control procedures for genome wide association studies. Curr Protoc Hum Genet 2011; Chapter 1: Unit 1.19. 10.1002/0471142905.hg0119s68 PMC3066182 21234875

[14] Purcell S, Neale B, Todd-Brown K, Thomas L, Ferreira MA, Bender D, Maller J, Sklar P, de Bakker PI, Daly MJ (2007). PLINK: a toolset for whole-genome association and population-based linkage analysis. Am J Hum Genet.

